# Multi-predictor mapping of soil organic carbon in the alpine tundra: a case study for the central Ecuadorian páramo

**DOI:** 10.1186/s13021-021-00195-2

**Published:** 2021-10-24

**Authors:** Johanna Elizabeth Ayala Izurieta, Carmen Omaira Márquez, Víctor Julio García, Carlos Arturo Jara Santillán, Jorge Marcelo Sisti, Nieves Pasqualotto, Shari Van Wittenberghe, Jesús Delegido

**Affiliations:** 1 Image Processing Laboratory (IPL), University of Valencia, 46980 Paterna, Valencia Spain; 2 Faculty of Engineering, National University of La Plata, B1900TAG La Plata, Argentina; 3 Faculty of Engineering, National University of Chimborazo, Riobamba, 060150 Ecuador; 4 Faculty of Forestry and Environmental Sciences, University of Los Andes, Mérida, 5101 Venezuela; 5 Faculty of Science, University of Los Andes, Mérida, 5101 Venezuela; 6 Faculty of Natural Resources, Higher Superior Polytechnic School of Chimborazo, Riobamba, 060155 Ecuador

**Keywords:** Carbon stock mapping, Soil organic carbon (SOC), Landsat, Random forest regression, Vegetation indices, Multispectral indices

## Abstract

**Background:**

Soil organic carbon (SOC) affects essential biological, biochemical, and physical soil functions such as nutrient cycling, water retention, water distribution, and soil structure stability. The Andean páramo known as such a high carbon and water storage capacity ecosystem is a complex, heterogeneous and remote ecosystem complicating field studies to collect SOC data. Here, we propose a multi-predictor remote quantification of SOC using Random Forest Regression to map SOC stock in the herbaceous páramo of the Chimborazo province, Ecuador.

**Results:**

Spectral indices derived from the Landsat-8 (L8) sensors, OLI and TIRS, topographic, geological, soil taxonomy and climate variables were used in combination with 500 in situ SOC sampling data for training and calibrating a suitable predictive SOC model. The final predictive model selected uses nine predictors with a RMSE of 1.72% and a R^2^ of 0.82 for SOC expressed in weight %, a RMSE of 25.8 Mg/ha and a R^2^ of 0.77 for the model in units of Mg/ha. Satellite-derived indices such as VARIG, SLP, NDVI, NDWI, SAVI, EVI2, WDRVI, NDSI, NDMI, NBR and NBR2 were not found to be strong SOC predictors. Relevant predictors instead were in order of importance: geological unit, soil taxonomy, precipitation, elevation, orientation, slope length and steepness (LS Factor), Bare Soil Index (BI), average annual temperature and TOA Brightness Temperature.

**Conclusions:**

Variables such as the BI index derived from satellite images and the LS factor from the DEM increase the SOC mapping accuracy. The mapping results show that over 57% of the study area contains high concentrations of SOC, between 150 and 205 Mg/ha, positioning the herbaceous páramo as an ecosystem of global importance. The results obtained with this study can be used to extent the SOC mapping in the whole herbaceous ecosystem of Ecuador offering an efficient and accurate methodology without the need for intensive in situ sampling.

## Background

Soil organic carbon (SOC) is the main component of soil organic matter (SOM), affecting essential biological, chemical and physical soil functions such as nutrient cycling, pesticide and water retention, and soil structure maintenance [1]. The accumulation of SOC is a slow process driven by environmental and climatic conditions. A sudden loss of SOC may therefore indicate soil degradation [2], land use changes or eventually also climate changes [3, 4]. An alteration of 10% in the total of SOC in the world’s soils is estimated to be equivalent to the anthropogenic CO_2_ emission over a 30-year time span [5]. Preserving the stock of SOC is therefore of global interest to the climate change mitigation and adaptation strategies [6]. Ecosystems with high amounts of SOC such as the Andean páramo, have a large potential to capture and store atmospheric CO_2_ [7]. The largest amount of SOC is typically stored into the top organic horizon layer of the soil (0 to 30 cm), and gradual decreases towards deeper soil profile sections [8–11]. These organic soils have high water retaining capacity, and in turn they accumulate water from thawing, rain, and fog condensation. The accumulated water is further released to the lowlands providing essential ecosystem services for the larger region of these ecosystems [7, 12]. Identifying such priority ecosystems and quantifying their SOC stock is a priority for the climate goals. Likewise, monitoring the SOC content of these priority carbon-rich ecosystems can provide vital information for making correct decisions regarding land uses at a global scale [13–16]. Since annual changes in SOC are considered small compared to the SOC stocks, such continuous monitoring of SOC could be at intervals of 5–10 years [8] in case robust prediction models are available.

The characteristics of these global SOC reservoirs are described by a set of soil biophysical and physical variables established by different fields [17]. Soil, vegetation and atmosphere characteristics, such as soil type and land use, together with climatic factors, land cover and topographic factors (including soil erosion) could be able to explain the dynamics of storage and the spatial distribution of SOC, being very useful especially in difficult geographical environments.

In the last years several methods have been used for SOC mapping, with the challenge to find the most appropriate and accurate one, based on study area characteristics and in situ data availability. Linear regression, geostatistical methods, and advanced nonlinear regression methods are used to predict the spatial distribution of the typically top layer soil properties [18]. Linear regression (simple and multiple) is used supposing a relationship between independent variables and the dependent variable under a linear form. Several studies used this method to estimate SOC, where the models explain up to 70% of the SOC variability [19, 20].

Geostatistical methods applied in soil mapping have the advantage of providing a statistically sound model for spatial variation, where the spatial autocorrelation is explicitly modelled and an explicit measure of the uncertainty is associated with the prediction [21, 22]. The limitations are due to the residuals which are assumed normally distributed, stationary and isotropic; in heterogeneous areas, the spatial variation models also fail to capture both gradual and abrupt changes in soil variation [22]. Therefore, in the large sample size the geostatistical models are computationally demanding [23]. Interpolations on data from in situ collected soil carbon such as ordinary kriging (OK) can be an appropriate and quick option [24], assuming no underlying trend in the data. When there is a spatial structure in the model residuals, hybrid models like residual kriging are applied to improve the SOC prediction performance [14, 25, 26]. Here, auxiliary variables are exploited combining geostatistics with additional information for SOC predictions [27]. Regression kriging (RK) and universal kriging (UK) also are applied for SOC mapping, where the improvement of RK techniques over OK largely depends on the strength of the correlation between SOC and the ancillary variables [28, 29].

As an alternative, for more complex soil-environment relationships it is also possible to use machine learning (ML) regression algorithms [30]. ML techniques are non-linear data-driven algorithms, where no assumption of the observations’ distribution is made. ML algorithms can also handle a large number of cross-correlated covariates as predictor variables [22]. Based on self-learning algorithms and supporting vector machines, ML techniques could further assist in the generation of even better calibration models for SOC prediction [10]. Also predictive tree models such as Classification and Regression Trees (CART) and Random Forest (RF) models [31] allow more accurate results and reduce the effect of noisy data [14, 32]. RF has demonstrated to provide reliable confidence intervals in SOC topsoil estimations [33]. In this context, RF models in combination with the appropriate selection of predictors can provide a powerful methodology for SOC mapping, applicable to both simple and complex geographical areas and when there a good data availability. Moreover, according to several authors adding new remote sensing predictors to the land cover analysis could greatly benefit the estimation of SOC distribution at diverse scales [10, 30, 34].

The soils of the Andean páramo ecosystem act as a large carbon sink due to its high capacity to retain SOC [35, 36]. It has an elevation gradient between 3000 and 4000 m a.s.l., maintaining a constant cold climate which reduces the mineralization of organic matter (OM) and produces large SOC reserves. Well-preserved soils in the ecosystem generally contain a larger amount of OM and therefore provide a greater storage capacity for carbon. Although the region is relatively well-conserved, human activities are present in the area, generally at lower elevations, where cattle is ranched and vegetation is often burned to grow pastures [37]. These changes in land use often produce significant losses in the soil carbon and reduce the capacity to further store carbon [38]. Despite the fact that studying the vast Andean páramo area and its changes on a regional scale is challenging due to the complexity given by its difficulty to access, the terrain irregularity, and the climatic conditions, the knowledge of this important carbon sink ecosystem is essential in the global attempt to quantify the SOC reserves and to make correct decisions on land uses [14]. However, digital SOC mapping studies for the region are scarce. In Ecuador, national-level knowledge of SOC reserves in this ecosystem is non-existent, and at the regional level it is limited or only punctually carried out [35]. The importance of herbaceous highland páramo ecosystems and their complex geography for sampling [36] have stimulated recent advances in methodologies for the estimation of SOC and its spatial distribution [39]. Finding good predictor variables for the spatial estimation of SOC which can be also remotely assessed is therefore considered of great scientific relevance.

In this respect, the objectives of this study are (1) to estimate and map SOC in the herbaceous páramo ecosystem based on a multi-predictor Random Forest (RF) regression technique applied to Landsat 8 (L8) satellite bands and indices in combination with complementary geographic information, (2) to identify the essential variables for SOC prediction, and (3) to evaluate the accuracy of SOC prediction in this complex mountain geosystem. To achieve these objectives, spectral bands and indices derived from the L8 sensors, OLI and TIRS, are combined with topographic variables from the digital elevation model (DEM), climate variables derived from the meteorological network in Ecuador and in situ collected SOC data to train and calibrate a self-learning algorithm with RF regression. The final step is the design of a SOC prediction model with a good level of accuracy used to map the spatial distribution of SOC in the study area.

## Materials and methods

### Study area

The study is carried out for herbaceous páramo ecosystem in the province of Chimborazo, Ecuador [40], 135 km south of the Quito city, and located in the central zone of the country. The ecosystem covers the largest area of mountain ecosystems in Ecuador, extending along the Andean mountain range, which borders the Western and Eastern Cordilleras arranged in meridian direction [7], from the province of Carchi to the province of Loja [41, 42].

**Fig. 1:**
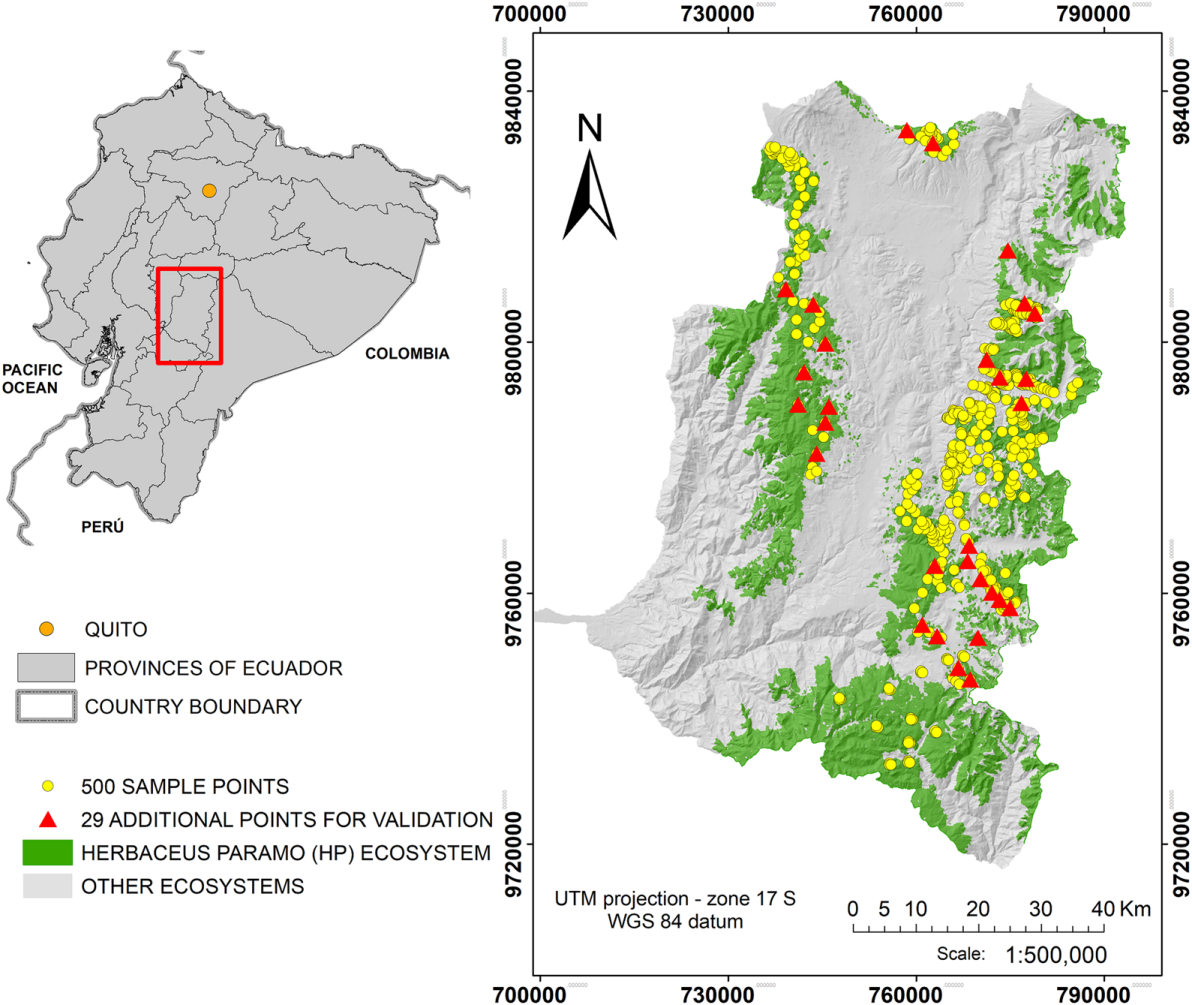
Distribution of the SOC sampling points in the study area location in Ecuador Chimborazo province

The herbaceous páramo is the largest subtype from sixteen subtype of páramo in Ecuador and cover above 75% approximately of the Ecuadorian páramo region [41, 42]. It extends between 78º39′ west longitude and 1º39' south latitude (Fig. 1). It is a mountainous geosystem with an irregular topography and elevation range between 2303 and 4501 m a.s.l, with a mean elevation of 3838 m a.s.l. Dry and wet seasons occur without notable differences, and the diurnal temperature change is most important as the annual changes of the mean temperature [43]. The mean annual temperature is 11 ºC and a high cloudiness is typical for this type of mountain ecosystem. The study area has 1667.6 km^2^, being 25.7% of the total province surface. The climatic and soil conditions are characterized by high humidity levels and high concentration of SOC. This results as a large amount of water per unit volume and an excellent water regulation capacity, between 0.55 and 0.90 cm^3^ cm^−3^ [7, 42, 44].

### Field data

In situ SOC data was collected between August 2016 and November 2017. Soil samples were taken from the top surface horizon layer (0 to 30 cm). Specifically, 500 points (for training and testing) were sampled with their respective geographical position (UTM coordinates zone 17S, WGS84) (PGS-Trimble JUNO SB handheld with 2-to-5-m positional accuracy in real time), and the SOC value was obtained in units of weight/weight % (g C/100 g of soil) and units of Mg/ha (Fig. 1). In order to carry out a post-validation of the model, 29 additional points were randomly sampled in July 2018 for a post-validation test, obtaining a total of 529 SOC points.

It is essential to have a large dataset to identify the ideal conditions that allow to find an excellent quantification of the SOC distribution over the study area. To find variables or indicators of SOC enables the possibility not only of obtaining a predictive model, as shown in the development of this article, but also, knowing the relationship between environmental variables with the storage dynamics of the SOC.

The SOC dataset (500 sample points for training and testing and 29 additional points of post-validation) is the result of a stratification of sampling units on the herbaceous páramo ecosystem, taking into account the geology and taxonomy of the soil available in the national system of geographic information [45]. Also, a review of the sampling units was made using the NDVI distribution (NDVI range between − 0.1 and 0.8, with a mean value around 0.4) to look at aspects of vegetation cover and variations, also an in situ survey of the sampling units was carried out as information to define the monitoring points. For that, topography, vegetation cover and the access to the entire ecosystem territory was considered, but the difficulty to access (ballast roads and some trails with steep slopes) was high or even impossible for some areas. Although we take the samples for training from August 2016 to November 2017, Wadoux et al. [46] confirmed the importance of the covariates used in the RF model more recently. Also, Wadoux et al. [46] have shown that the differences between prediction accuracies of different samples designs for large sample sizes become negligible.

For this reason, a random sampling was performed with a 95% confidence level. In situ samples were collected from 0 to 30 cm below ground using a blast-hole. Additional samples were taken at each site to determine the soil bulk density (in g/cm^3^), which was determined with 88 cm^3^ cylinders, taking undisturbed soil samples [47]. Soil samples were sieved (2 mm mesh), oven-dried at 105 °C for 24 h, and ground prior to analysis. The total SOC in the collected soil samples was determined with an Elemental Analyzer (Flash 2000 Organic Elemental Analyzer type CHNS/O, ThermoFicher Scientific). Specifically, a soil aliquot, containing approximately 10 mg of organic carbon in silver capsules, was weighed [48, 49]. The soil bulk density was calculated using the known volume cylinder method [50]. From the soil sample SOC weight % obtained (g C/100 g of soil) and the soil bulk density of the sample, the SOC content was expressed in Mg/ha [51].

### Satellite image processing

The multispectral images used in this study correspond to the Landsat-8 satellite (L8), obtained by the Operational Land Imager (OLI, bands 3 to 7) and the Thermal Infrared Sensor (TIRS, band 10 and 11) sensors. L8 images were downloaded from the Global Visualization Viewer (GloVis) web service of the United States Geological Survey [52]. The images used are from two L8 scenes, with approximately 75% of the study area located in the North scene, for which, the base image was LC80100612016325LGN01 (Table 1). It was also necessary to select two other scene dates for the gap filling, due to the large cloud cover in these base scenes. Based on a low annual meteorological variability (constant wet cold weather) typical of the páramo grasslands [7, 53], and the relatively stable SOC between consecutive years based on weather information available from the study area [45], images from the next year with similar dates were used for the filling on the North scene.

**Table 1:** Landsat-8 images used

L8 scene ID	Date	Use—path, row (WRS-2)	% CC^a^	Qlty^b^
LC80100612016325LGN01	2016/11/20	Base image (North scene)—010, 061	19.78	9
LC80100622016325LGN01	2016/11/20	Base image (South scene)—010, 062	25.59	9
LC80100612017263LGN00	2017/09/20	Filling—010, 061	32.44	9
LC80100612017023LGN01	2017/01/23	Filling—010, 061	57.94	9

^a^ Cloud cover (CC) percentage

^b^ Image quality for the bands (9 = best, 0 = worst, − 1 = not calculated) (Qlty)

The images were downloaded in GEOTIFF format, presenting a L1T pre-processing level, i.e., images with radiometric and geometric systematic correction by means of the incorporation of the ground control points [54] and ortho-rectification through the DEM. A verification was made by means of topographic maps and base cartography of rivers and roads, at a scale of 1:50,000, georeferenced in the UTM Datum WGS84 projection, from the Military Geographic Institute of Ecuador [55].

The downloaded satellite images contain the spectral information stored in digital numbers (DN) from the 16-bit L8 sensors to be converted to physical units such as radiance and reflectance. A radiometric calibration was applied through the Radiometric Calibration tool of the software ENVI 5.1 [56] to obtain radiance values at the top of the atmosphere (TOA). TOA radiance values were after converted to surface reflectance values by means of the FLAASH Atmospheric Correction tool also of ENVI 5.1, to remove the atmospheric dispersion based on two factors, (1) the radiance reflected by the canopy or earth surface to the sensor, and (2) the radiance that is dispersed by the atmosphere before arriving at the sensor.

Due to the climatic and atmospheric conditions, the mountainous páramo areas are highly affected by cloud presence. For this reason, the histogram of the images was analysed for cloud screening. Next, clouds were removed using the 16-bit Quality Assessment band to generate a mask, the Quality Assessment band is equal in size to the bands of the L1T product. This band has a decimal value at each pixel that represents the combinations of surface fill bits, atmosphere and sensor conditions that can affect the overall usefulness of a pixel [57].

Finally, the image mosaic was generated by means of the processed image of the scene LC80100612016325LGN01 that covers the north, center and part of the south zone of the study area and the processed image of the scene LC80100622016325LGN01 that covers the South zone. Cloud cover pixel gaps were filled in by the processed scenes LC80100612017263LGN00 and LC80100612017023LGN01 (Table 1).

Temperature was studied by means of the TIRS sensor [57], using the mean of bands 10 and 11. The top of atmosphere brightness temperature, TOA Brightness Temperature, is obtained in Kelvin (K) and converted to its corresponding value in degrees Celsius (^o^C). The annual variation in meteorological air temperature of the páramo is low [58], so, likewise the mosaic of the optical OLI sensor images, a mosaic of the TIRS band information was made with the images mentioned in Table 1.

### Spectral indices

Few studies use satellite sensors for SOC estimation [59]. However, the soil and the above-ground environment can be closely related allowing us to understand the biological, chemical and physical processes that govern the soil functions [60]. In recent years, however, SOC estimation and mapping based on remote sensing data are undergoing major developments including the use of new variable predictors [34]. In this regard, spectral indices can describe the surface characteristics in terms of vegetation cover, land use, and its changes. These characteristics can be further related to the soil properties and the soil type, such as soil moisture, and possibly establish covariance with SOC storage data [61, 62], by its influence into the global cycle of carbon [16, 63]. Different studies use the visible region considering that soils with higher carbon content have a darker appearance [63]. An extended literature review on remote sensing SOC estimates shows that different authors obtain good results in the 400–2200 nm spectral range [34, 64–68]. Here we use L8 OLI and TIRS spectral bands in the visible, near-infrared and thermal region (range 10.6 to 11.19 μm and 11.5 to 12.51 μm) to calculate spectral indices that are evaluated as possible indicators of SOC sequestration including indices related to the vegetation and soil moisture (NDVI, SAVI, WDRVI, EVI2, VARIg, NDMI), water (NDWI), bare soil (BI), snow cover (NDSI) and burnt soil (NBR, NBR2) in the case of the OLI sensor, and temperature in the case of the TIRS sensor (TOA Brightness Temperature). Table 2 shows the name, formulation and source of the spectral indices used.

**Table 2:** Established vegetation indices used in this study

Index/references	Formula	Formula with specific L8 bands
Normalized Difference Vegetation Index—NDVI [69]	$\mathrm{NDVI}=\frac{\mathrm{NIR}-\mathrm{R}}{\mathrm{NIR}+\mathrm{R}}$	$\mathrm{NDVI}=\frac{\mathrm{B}5-\mathrm{B}4}{\mathrm{B}5+\mathrm{B}4}$
Soil-Adjusted Vegetation Index—SAVI [70], L value according [71]	$\mathrm{SAVI}=\frac{\mathrm{NIR}-\mathrm{R}}{\mathrm{NIR}+\mathrm{R}+\mathrm{L}}\left(1+\mathrm{L}\right)$ $\mathrm{L}=0.15$	$\mathrm{SAVI}=\frac{\mathrm{B}5-\mathrm{B}4}{\mathrm{B}5+\mathrm{B}4+0.15}\left(1+0.15\right)$
Wide Dynamic Range Vegetation Index—WDRVI [72], a value according [71]	$\mathrm{WDRVI}=\frac{\mathrm{aNIR}-\mathrm{R}}{\mathrm{aNIR}+\mathrm{R}}$ $\mathrm{a}=0.05$	$\mathrm{WDRVI}=\frac{0.05\mathrm{ B}5-\mathrm{B}4 }{0.05\mathrm{ B}5+\mathrm{B}4}$
Enhanced Vegetation Index 2—EVI2 [73]	$\mathrm{EVI}2=2.5 \frac{\mathrm{NIR}-\mathrm{R}}{\mathrm{NIR}+2.4\mathrm{ R}+1}$	$\mathrm{EVI}2=2.5 \frac{\mathrm{B}5-\mathrm{B}4}{\mathrm{B}5+2.4\mathrm{ B}4+1}$
Normalized Difference Water Index—NDWI [74]	$\mathrm{NDWI}=\frac{\mathrm{G}-\mathrm{NIR}}{\mathrm{G}+\mathrm{NIR}}$	$\mathrm{NDWI}=\frac{\mathrm{B}3-\mathrm{B}5}{\mathrm{B}3+\mathrm{B}5}$
Visible Atmospherically Resistant Vegetation Index green—VARIg [75, 76]	${\mathrm{VARI}}_{\mathrm{G}}=\frac{\mathrm{G}-\mathrm{R}}{\mathrm{G}+\mathrm{R}}$	${\mathrm{VARI}}_{\mathrm{G}}=\frac{\mathrm{B}3-\mathrm{B}4}{\mathrm{B}3+\mathrm{B}4}$
Normalized Difference Snow Index—NDSI [77]	$\mathrm{NDSI}=\frac{\mathrm{SWIR}1-\mathrm{NIR}}{\mathrm{SWIR}1+\mathrm{NIR}}$	$\mathrm{NDSI}=\frac{\mathrm{B}6-\mathrm{B}5}{\mathrm{B}6+\mathrm{B}5}$
Bare Soil Index- BI [78]	$\mathrm{BI}=\frac{\left(\mathrm{SWIR}1+\mathrm{R}\right)-(\mathrm{NIR}+\mathrm{B})}{\left(\mathrm{SWIR}1+\mathrm{R}\right)+(\mathrm{NIR}+\mathrm{B})}$	$\mathrm{BI}=\frac{\left(\mathrm{B}6+\mathrm{B}4\right)-(\mathrm{B}5+\mathrm{B}2)}{\left(\mathrm{B}6+\mathrm{B}4\right)+(\mathrm{B}5+\mathrm{B}2)}$
Normalized Difference Moisture Index—NDMI [79, 80]	$\mathrm{NDMI}=\frac{\mathrm{NIR}-\mathrm{SWIR}1}{\mathrm{NIR}+\mathrm{SWIR}1}$	$\mathrm{NDMI}=\frac{\mathrm{B}5-\mathrm{B}6}{\mathrm{B}5+\mathrm{B}6}$
Normalized Burn Ratio—NBR [81]	$\mathrm{NBR}=\frac{\mathrm{NIR}-\mathrm{SWIR}2}{\mathrm{NIR}+\mathrm{SWIR}2}$	$\mathrm{NBR}=\frac{\mathrm{B}5-\mathrm{B}7}{\mathrm{B}5+\mathrm{B}7}$
Normalized Burn Ratio 2—NBR2 [82]	$\mathrm{NBR}2=\frac{\mathrm{SWIR}1-\mathrm{SWIR}2}{\mathrm{SWIR}1+\mathrm{SWIR}2}$	$\mathrm{NBR}2=\frac{\mathrm{B}6-\mathrm{B}7}{\mathrm{B}6+\mathrm{B}7}$
TOA Brightnees Temperature [57]	$T=\frac{\mathrm{K}2}{\mathrm{ln}\left(\frac{K1}{{L}_{\lambda }}\right)+1}$ T = TOA Brightness Temperature L_λ_ = spectral radiance K1 y K2 = thermal conversion constants	$B10, B11 TIRS sensor$

### Meteorological, topographical, geological and taxonomic data

#### Meteorological and climatic data

The SOC level in the soil is related to the amount of OM [83], which mainly depends on temperature, since at low temperatures biological activity is reduced, reducing the mineralization of the OM and allowing its accumulation in large quantities. Both temperature and precipitation are moreover determining the distribution and growth of vegetation making them very important control variables for both biomass growth and carbon storage in the soil [42].

**Fig. 2:**
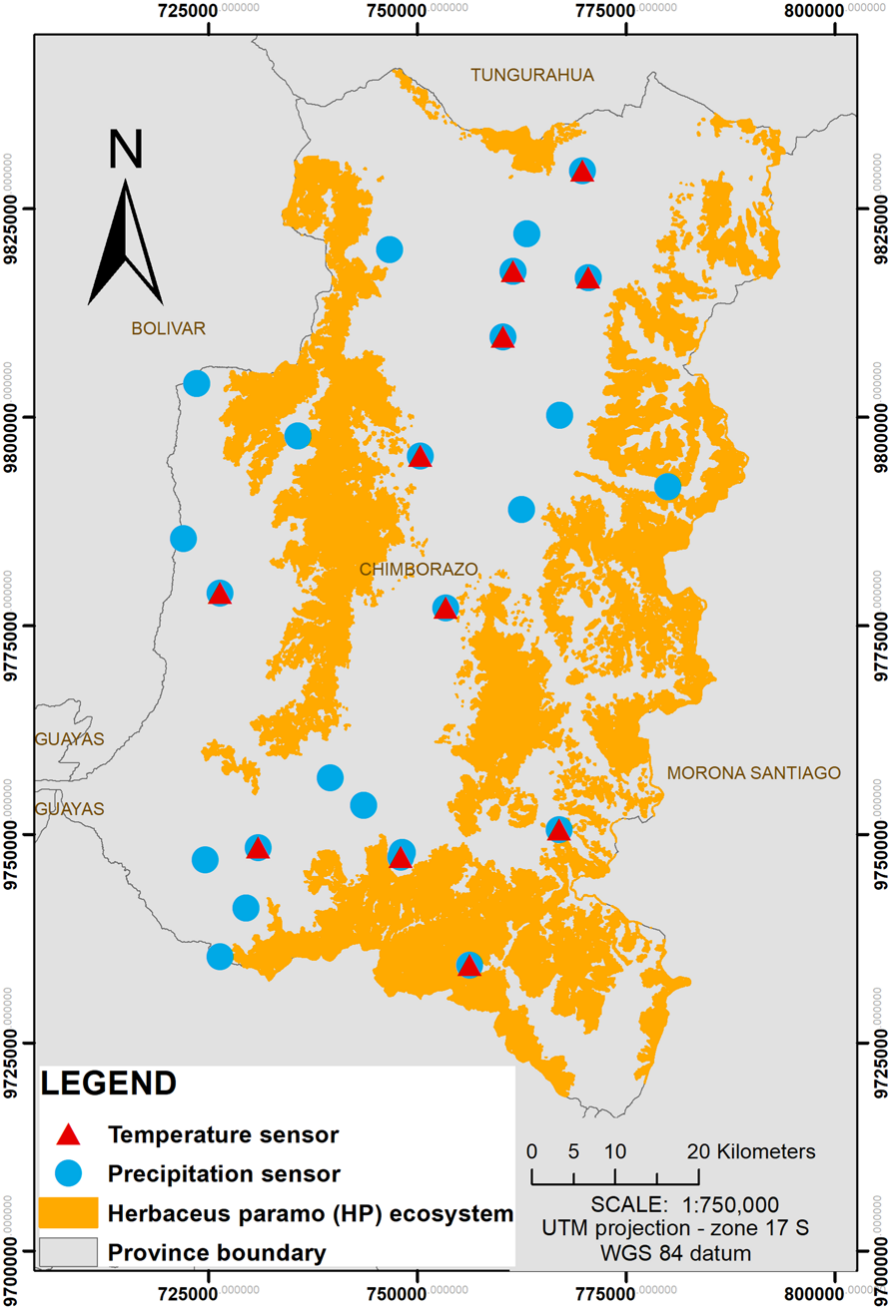
Location of weather stations with temperature and/or precipitation sensors

The temperature at the páramo generally decreases between 0.5 °C to 0.7 °C for each 100 m of elevation (from 2000 m a.s.l) [53]. Data on air temperature and precipitation are collected by the weather stations in the area (Fig. 2). The seasonal trend in temperature and precipitation data of the study area in practically equatorial territory show only small alterations in time, indicating humid and cold conditions with small fluctuations < 2 °C on a yearly basis, with month average temperatures commonly low 10 °C [40]. Therefore, it was decided to use the average annual air temperature data, as well as the total precipitation for the 2015 year, from the meteorological stations closest to study zone, taking into account the availability, quantity and quality of the data. The data from meteorological stations located within the Chimborazo province is provided by the National Institute of Meteorology of Ecuador (INAMHI, 21 stations), the data from the southeast of the Chimborazo province by the National University of Chimborazo (UNACH, 3 stations) (Fig. 2), and additional data from 33 meteorological station belonging to INAMHI located in surrounding areas out of the study area. Spatial interpolation methods are used to obtain weather information at a particular place where it can’t be attained directly [84]. Ordinary kriging stochastic interpolation method is widely used for spatial interpolation of meteorological data [24, 85]. We generated distribution layers of the surface variables through geostatistical analysis with the OK method and with a semivariogram adjustment taking into account the spatial variation of relationships between stations [84]. Most part of the study zone has an average annual temperature between 8 and 14 °C, with minimum of average temperature of 5 °C and a mean around 11 °C. The annual accumulation of precipitation in the study area has a minimum and maximum of 390 mm and 2380 mm, respectively.

#### Topographic data

Soil erosion can alter and change the SOC by causing a significant loss of relatively stable and long-term stored SOC in the top soil layers [86]. From the DEM, the slope and orientation variables were analyzed and, in order to evaluate the effect of soil erosion, the dimensionless Slope Length and Steepness Factor (LS Factor) variable was calculated [87].

The LS Factor describes the topographic effect on the soil erosion, and it incorporates the potential for soil erosion due to surface runoff. This is based on two factors; the factor L informs about the impact of the length of the slope while the factor S explains the effect of the slope's inclination. This factor is appropriate for estimating landscape erosion in complex topographies. The procedure was performed in ArcGis 10.2 software applying Eqs. 1, 2, 3, 4, 5 and 6 [88].

1 $$LS=L*S$$

with the L factor calculated as:

2 $$\beta =\frac{\frac{\mathrm{sin}\theta }{0.0896}}{{3\mathrm{sin}\theta }^{0.8}+0.56}$$

3 $$m=\frac{\beta }{\beta +1}$$

4 $$L=\frac{{\left[{A}_{i,j}+D\right]}^{(m+1)}-{{A}_{i,j}}^{(m+1)}}{{x}^{m}{D}^{m+2}{(22.13)}^{m}}$$

where A_i,j_ is the accumulation area with coordinates (i,j) [m^2^]; D is the length of the pixel size [m]; x is the shape coefficient [dimensionless]; m has values between 0 and 1 [dimensionless]; $\theta$ is the slope angle [rad]; and $\beta$ is the ratio of rill to interill erosion [dimensionless].

The S factor is calculated as:

5 $$S=10.8\mathrm{sin}\theta +0.03 ,\quad \mathrm{if}\, tg \, \theta < 0.09$$

6 $$S=16.8\mathrm{sin}\theta -0.05 , \quad \mathrm{if} \, tg \, \theta \ge 0.09$$

#### Geological and soil taxonomic data

**Fig. 3:**
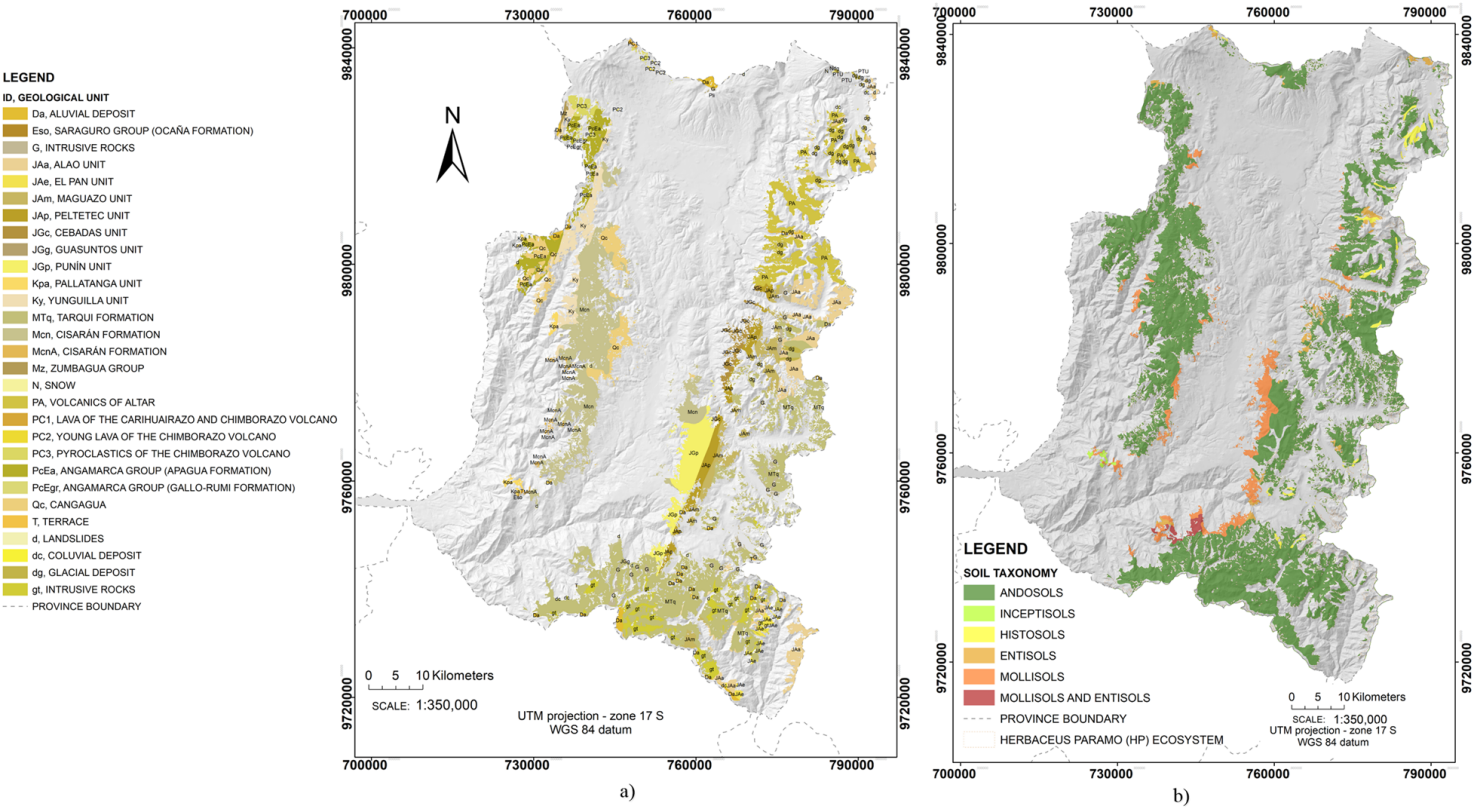
**a** Geological unities map of the páramo herbaceous ecosystem; **b** Soil taxonomic map under the páramo herbaceous ecosystem in the study area

The geological classification of the soil is based on its origin and evolution over time. The national geological mapping classification was used [45] containing 31 geological unities with names referring to the site locations (Fig. 3a).

There are soils of volcanic origin, which would have a high content of OM. On the outer slopes there are perennial forested areas, often with thick cloud cover present. These soils are partially covered by recent volcanic ash and rejuvenated by the soil erosion. There are also soils with sediments of recent and long-standing volcanic origin. Similarly, alluvial soils with some agricultural activity occur [7]. These characteristics and geological conditions in the páramo herbaceous soils may favor the storage of SOC in high quantities.

Further, the variable layer of the soil taxonomy was included. According to this variable, the soil is considered as a natural body comprising solids (minerals and OM), liquids and gases on the land surface. It is characterized by horizons or layers distinguished from the initial material as a result of additions, losses, transfers and transformations of energy, also by the ability to support plants in a natural environment. To categorize the soil based on their taxonomy, the USDA Soil Taxonomy was used, recognized in Latin America [89]. The soils in the study area include predominately Andosols, together with Entisols, Histosols, Inceptisols and Mollisols (Fig. 3b). Due to the volcanic origin of these soils, they have high OM and SOC content, differences in SOC stock capacity can be evaluated based on this taxonomy variable.

### SOC prediction with Random Forest Regression

**Fig. 4:**
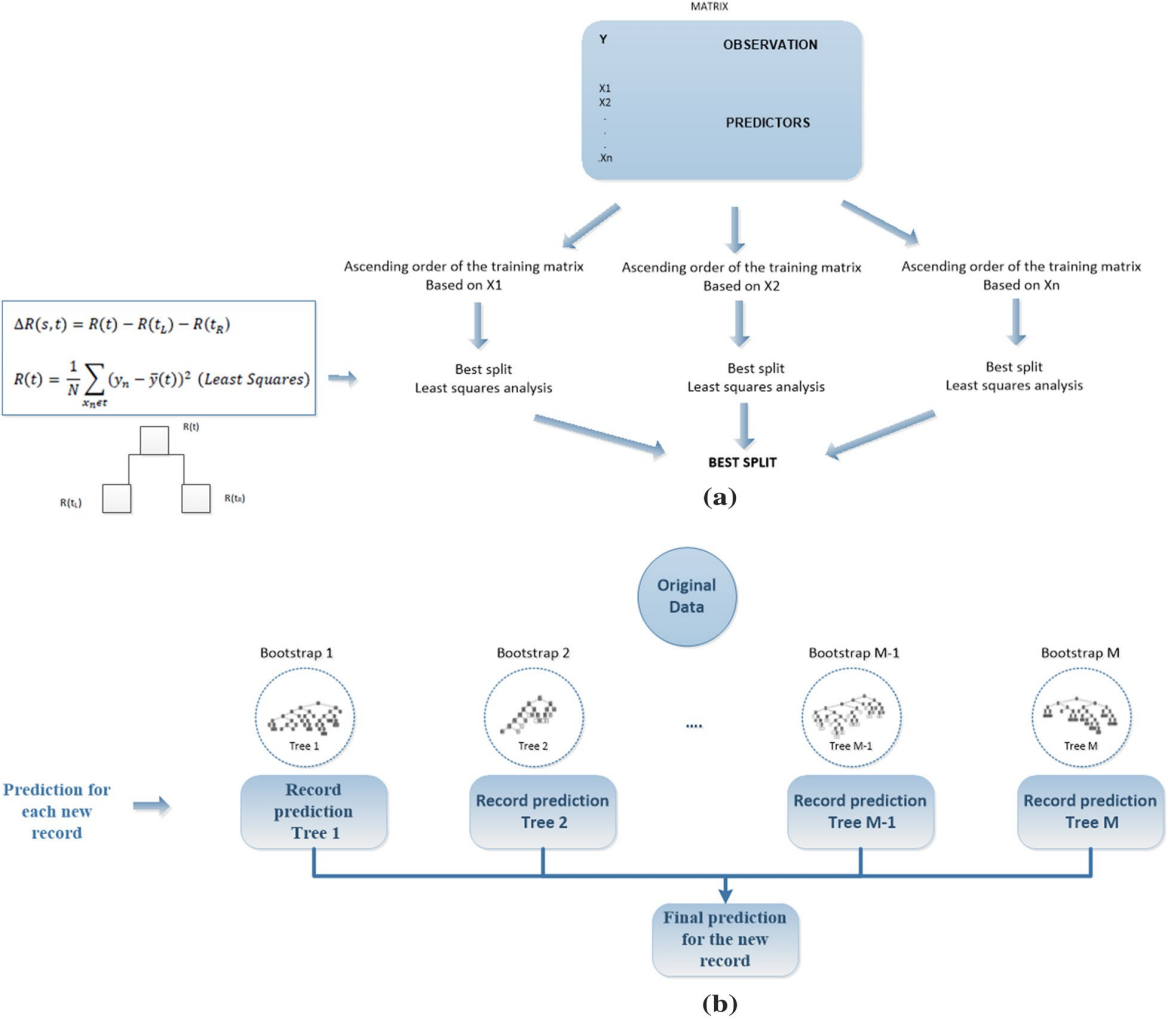
**a** Process followed by a CART algorithm to find the best split; **b** Diagram of the process carried out by the RF regression algorithm. Based on [31, 91, 92]

For the SOC prediction Random Forest (RF) regression was used (Salford Systems software, version SPM8.2). RF is a combination of tree predictors and formed by growing a tree structure depending on a random vector while the tree predictor takes on numerical values [31]. RF regression was performed to obtain the quantitative value of SOC, with taking numerical values instead of class labels as tree predictors and the training set is independently extracted from the random vector distribution. One of the advantages of RF is that it is robust despite the presence of noise, i.e. the presence of anomalous data [31]. The algorithm is based on classification and regression trees (CART) [90, 91]. The CART finds the variables with predictive possibility and the division points that reduce the quadratic and absolute error in order to predict the dependent variable SOC with greater accuracy. CART evaluates all possible predictors and all possible split points for each predictive variable and determines the best split for each one, making a comparison between the best splits of each possible predictor and choosing the split based on the standard deviation of each option. In Fig. 4a, the process followed by a CART algorithm to find the best break or split point in the tree is shown.

Therefore, the RF regression algorithm adjusts multiple CART trees to independent sample bootstrap data and then combines the predictions. A bootstrap is a random sample for replacement purposes, it is created by randomly selecting one record at a time from the original data, an observation can be chosen more than once. The process is carried out until the same number of records of the original data are completed. A CART tree is created in each bootstrap, and only K variables selected randomly are considered in each partition of the tree instead of all of them. The process is performed M times according to the number of bootstraps created. At the end a prediction record for each tree is obtained, and the final prediction is the result of the average of M predictions. The performance of the algorithm depends on parameters such as K and M, set in this study. In this way the RF prediction is an average of the prediction of the CART trees created in each bootstrap (from 1–M) (Fig. 4b). Each of the records of the matrix are submitted down each bootstrap decision tree, generating at the end of its step a prediction based on the bootstrap CART (1–M).

### Model training, optimization and calibration process

**Fig. 5:**
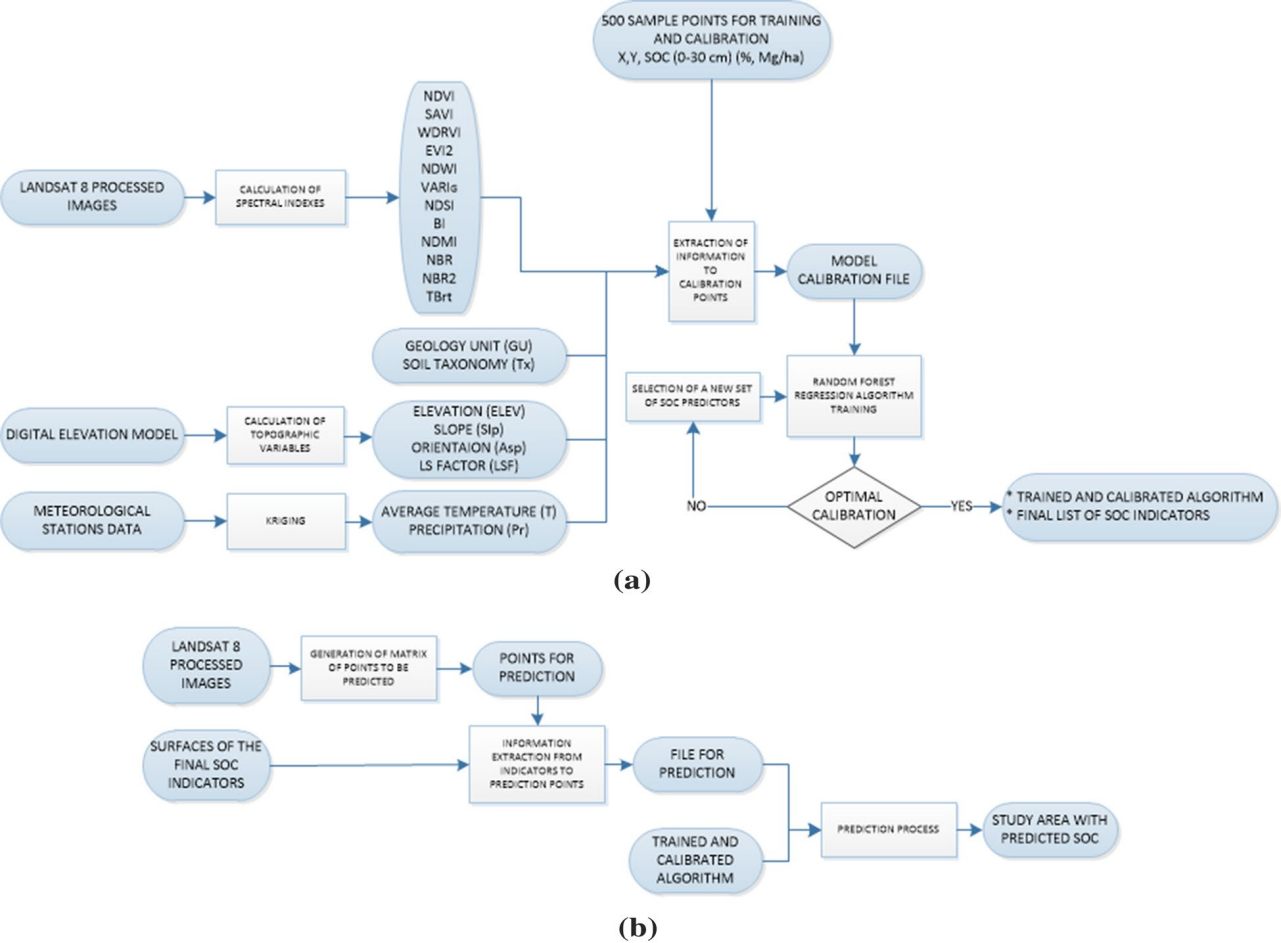
**a** Methodology for generating the SOC prediction model through the Salford Systems software SPM8.2; **b** Application of the trained and calibrated SOC algorithm for SOC pixel-based prediction mapping in the study area

The methodology used to calibrate the RF regression algorithm for the prediction model is summarized in Fig. 5a. The training and calibration dataset was created based on the extraction of all prediction variables to the 500 in situ SOC sample X, Y coordinates. 315 SOC samples were used for training and 185 for calibration Out of Bag (OOB). The training of the prediction model uses sets of variables based on different combination ways to evaluate their predictive functionality. In total 20 variables are evaluated as possible SOC predictors in this study (see Table 3), including spectral variables (EVI2, WDRVI, SAVI, NDVI, NDWI, VARI_G_, NDSI, BI, NDMI, NBR, NBR2, TOA Brightness Temperature (TBrT)), topographic variables (elevation, LS Factor, slope and orientation), climatic variables (average annual air temperature and precipitation), and variables corresponding to Ecuador's soil base mapping (geological unit and soil taxonomy). The use of the variables was jointly and thus also through combined subgroups, to rule out non-useful variables and to optimize the model. The model calibration starts with all variables (20). Then, variables with less relative importance are discarded, these variables provide limited information to improve the model. The number of tree nodes used is adjusted when the RMSE is stable, the final model is obtained when the maximum determination coefficient (R^2^) was reached (Eqs. 7, 8 and 9). The Mean Squared Error (MSE) (Eq. 10), Mean Absolute Deviation (MAD) (Eq. 11) and Mean Absolute Percentage Error (MAPE) (Eq. 12) also were observed.

**Table 3:** Characteristics of variables evaluated as possible SOC predictors

Type of variable	Variable	Spatial resolution/scale	Source
Spectral	Indices: EVI2, WDRVI, SAVI, NDVI, NDWI, VARIG, NDSI, BI, NDMI, NBR, NBR2	30 m	Landsat 8 images—OLI sensor (see Table 1) [57]
Spectral	TOA Brightness Temperature (TBrT)	100 m	Landsat 8 images—TIRS sensor (see Table 1) [57]
Topographical	Elevation, LS Factor, Slope, Orientation	30 m	DEM Ecuador-SNI Ecuador [45]
Climatic	Average annual air temperature, precipitation	-	Interpolations using data of meteorological stations (54 stations belonging to INAMHI and 3 belonging to UNACH)
Geological and soil taxonomic	Geological Unit, Soil Taxonomy	1:50,000	Geology, taxonomy of Ecuador-SNI Ecuador [45]

7 $${R}^{2}=1-\frac{SSE}{SSY}$$

8 $$SSY=\sum_{i}^{n}({{y}_{i}-\overline{y })}^{2}$$

9 $$SSE=\sum_{i}^{n}({{y}_{i}-{f}_{i})}^{2}$$

10 $$MSE=\frac{SSE}{n}$$

11 $$MAD=\frac{\sum_{i}^{n}{|y}_{i}-{f}_{i}|}{n}$$

12 $$MAPE=\frac{100}{n}\sum_{i}^{n}\frac{{|y}_{i}-{f}_{i}|}{{f}_{i}}$$

where SSY is the total sum of squares; and SSE is the sum of squares of residuals; ${y}_{i}$ is the observed value; ${f}_{i}$ is the predicted value; $\overline{y }$ is the mean of the observed data; and n is the number of sample points.

Concretely, the accuracy of each SOC prediction model is obtained after the algorithm calibration based on the OOB dataset, which are about one-third of the data-points left out conforming the OOB for each bootstrap [31]. Each OOB record is subjected to the algorithm as it passes through its decision trees and its accuracy is recorded. Similarly, the OOB dataset is used to evaluate the importance of each predictor variable to optimize the model, obtaining the precision and an indicator of the variable importance for the model [71]. Each variable is evaluated individually considering the decrease in precision when erroneous values are entered, then the variables are rescaled to have values between 0 and 100 (for each variable). The most important variable is given 100% while the rest of variables are relatively expressed to this one [31].

To predict the SOC for each pixel, the spatial resolution of the satellite images was used to create a homogeneous spatial distribution of points in the study area, and with their respective associated coordinates (X, Y), forming the prediction file. Then, predictors data values are extracted into the prediction file. Each record is evaluated into the model of SOC prediction generated, finally the quantitative value of SOC content for each pixel in the study area is predicted (see Fig. 5b).

In addition to the calibration of the predictions validated through OOB, a final independent validation process was carried out with additional in situ data not used in the training/calibration and not used on the OOB. For this purpose, post-validation sample points were used (29 additional points, detailed in Sect. 2.2), obtaining the real value of SOC through a laboratory analysis. Twenty-nine validation points collected in 2018 were used, by which the accuracy between the predicted model and in situ value was evaluated based on the root mean square error (RMSE).

## Results

**Fig. 6:**
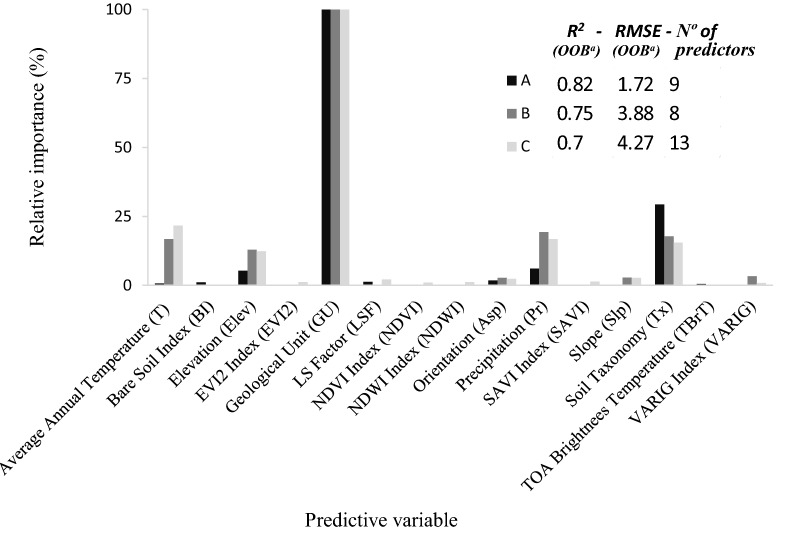
Relevant model calibration results in SOC (in weight %) prediction. Relative importance of the models’ prediction variables is shown, giving 100% to the most important predictor and relative importance (in %) for each other variable in the respective model [31]**.** Statistical performance indicators are shown. ^a^ OOB testing data (185 samples)

Multiple model optimization and calibration tests were carried out, evaluating each spectral, meteorological, topographical, geological and taxonomic variable, in order to find the optimal calibration and final list of SOC predictors. This is a long process that starts using all variables (20), then each non-contributing variable to improve the model is discarded, until the best model and combination of variables is found. Figure 6 shows the results of the three most relevant models, with the highest determination coefficients (R^2^), and at the same time the lowest RMSE. The algorithm sorts the evaluated variables according to their relative importance so that the user can locate and choose to discard one or more variables. Tree nodes between 400 and 1000 was probed, with 400 tree nodes the R^2^ value was low and the RMSE is not stable. With 1000 tree nodes a highest value R^2^ was obtained with a stable RMSE, with 500 tree nodes the results were the same, so in order to optimize the model on this study 500 tree nodes were used.

The best performing models with highest accuracy are presented on Fig. 6. The predictors GU, T, Pr, Tx, Elev and Asp are common on the three models (A, B and C). The model A was based on 9 predictors noticeably improved the model, reducing the RSME and reach a R^2^ of 0.82. In the models B and model C the R^2^ decrease, variables such as NDVI, NDWI, SAVI, EVI2 vegetation indices decreased the performance of the model (model C). Variables such as VARIG, SLP, NDVI, NDWI, SAVI, EVI2, WDRVI, NDSI, NDMI, NBR, NBR2 were discarded due to their minor statistical relevance for the prediction. These variables do not have a strong link established with SOC. While other studies have shown results with NDVI index as a SOC predictor [93–95], the index is shown less relevant for the complex Andean ecosystem.

**Fig. 7:**
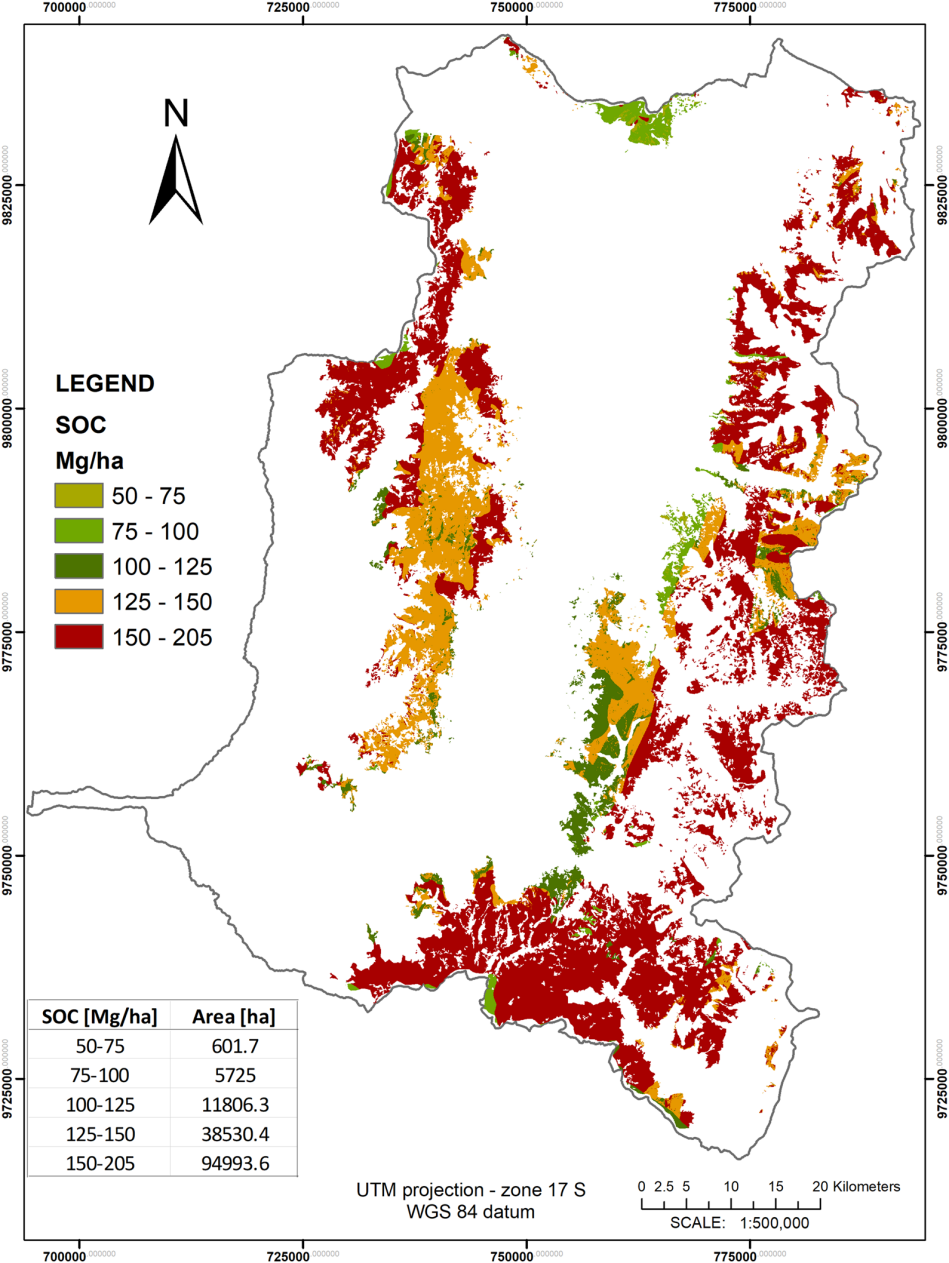
Result map of SOC prediction (in Mg/ha) in the 0–30 cm profile

The correlation matrix of model A predictors variables is shown in Table 4. Where, a natural correlation between SOC (in weight %) and SOC (in Mg/ha) is observed, the correlation between SOC with Soil Taxonomy and Precipitation also stands out. On the other hand, there is a negative correlation between SOC and Average annual temperature. Figure 6 indicates the relative importance of the predictive variables according to their contribution to the performance of the SOC prediction in model A (see advanced statistics in Table 5). For 185 OOB testing data points, a RMSE of 1.72% and a R^2^ of 0.82 for the predictive model of SOC % were obtained. SOC in Mg/ha is not proportional to SOC %, as the unit conversion depends on the soil bulk density, so the model A was used but with the SOC dataset values in units of Mg/ha. The results were a RMSE of 25.8 Mg/ha and a R^2^ of 0.77. Finally, the SOC mapping in the profile 0–30 cm, was obtained in units of % and then in units of Mg/ha see Fig. 7.

**Table 4:** Correlation matrix based on Pearson correlation coefficient of model A predictors

	Elev	Tx	GU	LSF	Asp	BI	TBrT	Pr	T	SOC %	SOC Mg/ha
Elev	1.00										
Tx	− 0.11	1.00									
GU	0.24	− 0.28	1.00								
LSF	− 0.30	0.06	− 0.29	1.00							
Asp	− 0.15	− 0.13	0.07	0.08	1.00						
BI	0.32	− 0.14	0.27	− 0.28	− 0.11	1.00					
TBrT	− 0.09	0.02	0.16	− 0.23	− 0.29	0.46	1.00				
Pr	− 0.12	− 0.33	0.17	− 0.17	0.05	0.18	0.17	1.00			
T	− 0.01	0.18	− 0.24	0.15	0.004	− 0.15	− 0.18	− 0.47	1.00		
SOC %	0.001	− 0.43	0.25	− 0.05	0.09	0.12	-0.003	0.50	− 0.26	1.00	
SOC Mg/ha	0.02	− 0.30	0.20	− 0.05	0.07	0.11	0.01	0.39	− 0.17	0.88	1.00

**Table 5:** Advanced statistics for model prediction of SOC (Model A)—Model error measures from the OOB testing data

	RMSE	R^2^	MSE	MAD	MAPE
SOC %	1.72	0.82	2.96	1.20	0.14
SOC in Mg/ha	25.78	0.77	664.82	18.60	0.17

Geological unit, soil taxonomy, precipitation and elevation were the variables that provided the most information in the SOC prediction model. This is in line with the studies of dynamics in SOC and its decomposition [96–99], where, environment and soils properties like soil texture and structure, precipitation, and average temperature have a great impact. To identify the SOC variations is a difficult task, in particular on sites without human intervention. The SOC variations can be related to variables from biological and pedogenic processes [100], explained by the high importance of geological unit and soil taxonomy. In short, it was possible to find that the variables that drive the SOC prediction model maintain a physical–chemical relationship with the dynamics of carbon sequestration [8, 98], where, the soil geology, the composition, soil properties, surface soil conditions and topographic factors as well as the climate environment play a very important role. Also, it allows us to know the degree of importance of each variable around the amount of SOC prediction and sequestration (see Fig. 6).

One spectral index, the Bare Soil Index (BI), was found relevant in the prediction model, with high values of BI indicating a decrease of SOC content. The same effect was noted with values of the soil erosion factor (LS Factor). It can explained by the fact that when the better conserved the soil, it has more organic matter, better carbon storage and better water regulation [101]. Also soil erosion by wind and water, subsequent sediment transport, and depositional processes may lead to soil organic carbon (SOC) loss [86]. Since the scale of the variables geological unit and taxonomy have a rather low spatial resolution, predictors variables such as BI index derived from satellite images and derived from DEM could increase the resolution of the spatial prediction. However, these variables show only a minor relative importance to the main variable, 1.17 and 1.32%, respectively.

Figure 7 shows the SOC map obtained from Model A, showing that the largest percentage (over 57%) of the study area, contains high concentrations of SOC, between 150 and 205 Mg/ha and lowest SOC values were between 50 and 75 Mg/ha, principally on Andosols and Inceptisols soils. The high SOC content found is larger compared to SOC content values in pristine soils in other world areas, while the low SOC content values are similar to the content values in intervened soils [102]. Processes related to erosion, decomposition and leaching decrease the SOC content [86, 103].

**Fig. 8:**
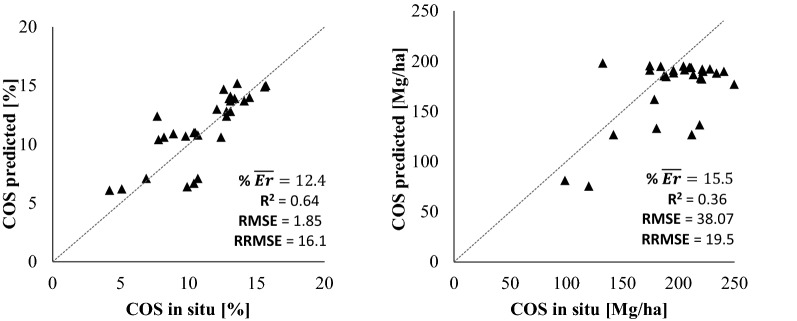
Validation of the Model A prediction model for SOC expressed in weight % (left) and in Mg/ha (right) based on an independent randomly sampled dataset (29-points). The 1:1 line is shown

An independent validation test of the Model A with the additional dataset of 29 points is performed. Figure 8 shows the validation statistics for both SOC predictions in % and Mg/ha for the 29-points. Through the additional field sampling performed, it can be observed that the results of both models are good, although the SOC (%) based prediction model has less error. This could be explained due to the complexity to account for the soil bulk density by the prediction model. Soil bulk density is a relevant property to obtain more correct SOC values in Mg/ha on an area basis [8, 50, 51], but a uncertainty error by the soil bulk density can be added [104]. Figure 8 resumes the results based on errors values, obtaining fairly good values of relative RMSE in both cases (16.1 and 19.5%). Also, we can note that high concentrations of SOC would be more difficult to predict. It would be explained due to the soils with high in organic carbon are also the most affected by the variability in soil bulk density [105].

## Discussion

The results of the RF models selected expose the most relevant SOC predictors from a group of 20 variables, carefully chosen for their possible relationship with SOC sequestration. A model with nine variables predicting SOC storage was calibrated with a good accuracy. The most important SOC predictors from the model are given by geological unit, soil taxonomy, precipitation and elevation. This was expected, considering the well-documented classes of soils present in the herbaceous páramo ecosystem based on the Geological unities (31) and the soil taxonomy classification (Andosols, Entisols, Histosols, Inceptisols and Mollisols). Environmental factors on the land surface moderates the exchange of water, energy, and greenhouse gases between the land and the atmosphere [106, 107]. In the páramo region the dynamic SOC storage is known to linearly increase with precipitation intensity [108]. A higher precipitation provides moisture in soils and contributes in its amount of water retention, influenced by the type of soil and vegetation, among others. Jenny [106] and Adhikari et al. [107] found that topographic variables had a higher influence at finer scales, whereas climatic variables were more important at coarser scales. Therefore, due to the large study area of 1667.6 km^2^ and large elevation range between 2303 and 4501 m a.s.l, it is possible to explain the importance of elevation and another topographic predictors for the resulting model.

Other studies on SOC estimations provided equal value ranges and accuracies. A SOC case study in Cameroon, on 3 horizons (0–15 cm, 15–30 cm and 30–100 cm) of soil used a hybrid machine learning modelling and legacy soil data, provided R^2^ values between 0.52 and 0.67 for SOC ranges between 11 and 210 Mg/ha at the 0–30 cm horizon. In the case of study areas with large elevation ranges, the elevation and the weather variables precipitable water vapor and rain are typically linked with SOC [109]. In a Chinese case study with great variety of vegetation and soil types and distribution patterns, 67% of SOC variation was explained by DEM and NDVI parameters through an artificial neural network combined with kriging (ANN- kriging), and regression tree (RT) models [28]. A north American case study in the state of Indiana showed the highest prediction accuracy (R = 0.75) using ordinary kriging over a study area with low variation in elevation [24]. In general, hybrid spatial models applied based on ML algorithms including auxiliary variables (slope, elevation, Topographic Wetness Index (TWI), among others) could increase the accuracy of predictions, but if the selection of variables is not appropriate for the study area model predictions might be rather poor [27].

Including remote sensing techniques for the prediction of SOC has led to poor to good results (R^2^ = 0.23–0.67) [34], where spectral information from Environmental Mapping and Analysis Program (EnMAP) and Sentinel-2 sensors have shown the best results [67, 68]. In this work, L8 imagery was used and processed to correct and eliminate clouds cover, obtaining spectral information for the entire study area, without the necessity of eliminate sections for cloudiness concept. The rich database used in this study, with 500 SOC data point trying to encompass all soil heterogeneity, made it possible to obtain enough information to calibrate the RFR model with high accuracy. Moreover, terrain knowledge of the study area was fundamental to prioritize the election of variables to be evaluated as a SOC predictors.

Several remote sensing techniques that varying depending on their spatial, spectral, temporal and radiometric resolution and the platforms that are mounted on (spaceborne platforms, airborne platforms and unmanned aerial systems) can help to quantify the soil carbon sequestration [34]. Gholizadeh et al. [110] found that Red and NIR bands, also spectral indices BI, SAVI among others provide the strongest correlations with SOC. Which agrees with the results of this study, where the BI was found as SOC predictor, so too the TOA Brightness Temperature.

Only few SOC studies focus were performed in the páramo region [39, 111]. Due to the soil chemistry of these alpine tundra ecosystems they are known to contain very high carbon in the soil [7, 58], but predicting the SOC stock and its distribution remains a complex task. A large sampling density is in favor for RF regression for SOC mapping but due to some limited access of certain areas a homogeneous sampling over the entire herbaceous páramo was not possible. This was the reason to use a stratification of sampling units as a sample design, avoiding sampling points in the edges, to reduce errors by delimitation of stratification or precision by GPS. The limitation on this sampling design is the lower density of sample data points within zones difficult to access. On the other hand, areas with less accessibility are less exposed to anthropic interventions, therefore SOC alterations in these areas are expected to be low.

Erosion, transport, and depositional processes redistribute landscape SOC [86], whence in this study, the topographic factor and the use of remote sensors were widely analyzed to decrease errors by produced by these environmental phenomena in the model. Further, this study analyzed the link and predictive power of soil (cover) variables, using remotely sensed vegetation indices, including also the TOA Brightness Temperature, in addition to classic SOC predictors. Combined with L8 (OLI and TIRS sensors) spectral data, the RFR model based on a wide set of climatologic, geophysical and biophysical predictor variables resulted in good predictions.

A future extension of this work would be to use other satellite sensors such as Sentinel-2 [112], based on the automatic products such as Leaf Area Index (LAI), Fractional Vegetation Cover (FVC), Leaf Chlorophyll Content (Cab) and Canopy Water Content (CWC) among others. Hence, biophysical variables, available at higher spatial resolution, could be further used to explain a possible linking with the SOC storage capacity of the herbaceous páramo soils.

## Conclusions

In this research, a soil organic carbon multi-predictor model for the complex Andean páramo area was calibrated with an accuracy level of 82% for SOC in weight % and 77% for SOC in Mg/ha. The spatial estimation of SOC is challenging to achieve for complex areas due to effects of climate, topographical variability and geological diversity, among others. By optimizing a Random Forest (RF) automatic learning algorithm, nine environmental variables related to the dynamics of SOC sequestration were selected. The variables geological unit, soil taxonomy, precipitation, height, orientation, LS factor, BI index, average annual temperature and TOA Brightness Temperature were found to have great relevance in the quantification of SOC.

The prediction of SOC is strongly driven by the geological unit and the soil taxonomy, followed by the mean yearly precipitation and elevation. Considering the terrain topographical complexity to be studied, as well as the geological heterogeneity, variables with better spatial resolution may improve the resolution for SOC distribution results. This is the case of the variables obtained through the OLI and TIRS remote sensors. Only a few spectral indices were important for SOC prediction model, i.e. the BI index and the TOA Brightness Temperature. Nonetheless, due to the heterogeneity of the study area, predictors with less relative importance for the model such as the BI index, orientation, LS Factor, average temperature and TOA Brightness Temperature helped to increase the accuracy of the RF regression model.

The use of remote sensing data for the mountainous páramo ecosystem, was challenging due to extreme weather conditions causing high percentages of cloudiness in the satellite images. For this reason, the time dedicated to image selection, treatment, and detection of cloud areas, as well as filling in was a laborious, meticulous and a critical process. Even so, the methodology used in image processing and geographic information generation was useful and it was possible to achieve valuable information for the model. The next research step for these complex environments would be to consider how to introduce alternatives where the atmospheric influence can be minimized, as is the case with RADAR—Radio Detection and Ranging sensors.

The proposed methodology could be used by research groups studying similar complex ecosystems, as the high Andean areas. The results of the SOC quantification and mapping in the herbaceous páramo ecosystem are of vital importance to the global objective of knowing and quantifying the SOC reserves. The high SOC values further demonstrate the importance of knowing the SOC reserves in these ecosystems, including the factors which control or affect the mineralization process on the one hand, and the soil degradation effects due to erosion, on the other hand. Moreover, the results obtained through the digital mapping of the high reserves of SOC represent a great contribution in the soil characterization of the Ecuadorian territory. This will allow to establish provincial and national regulations to prevent soil degradation in this type of ecosystem, given their importance in soil structures stabilization, water regulation and carbon storage. Such actions will further allow a better conservation and management of these valuable carbon storage ecosystems from a local towards a global perspective.

## Data Availability

The datasets during and/or analyzed during the current study available from the corresponding author on reasonable request.

## Abbreviations

SOC: Soil organic carbon
SOM: Soil organic matter
OM: Organic matter
OK: Ordinary kriging
RK: Regression kriging
UK: Universal kriging
ML: Machine learning
OLI: Operational Land Imager
TIRS: Thermal Infrared Sensor
CART: Classification and Regression Trees
RF: Random Forest
L8: Landsat 8
DEM: Digital elevation model
GloVis: Global Visualization Viewer
DN: Digital numbers
TOA: Top of the atmosphere
NBR2: Normalized Burn Ratio 2
SAVI: Soil-Adjusted Vegetation Index
WDRVI: Wide Dynamic Range Vegetation Index
EVI2: Enhanced Vegetation Index 2
NDWI: Normalized Difference Water Index
VARIg: Visible Atmospherically Resistant Vegetation Index green
NDSI: Normalized Difference Snow Index
BI: Bare Soil Index
NDMI: Normalized Difference Moisture Index
NBR: Normalized Burn Ratio
NBR2: Normalized Burn Ratio 2
LS Factor: Slope Length and Steepness Factor
TWI: Topographic Wetness Index
RMSE: Root mean square error
R2: Determination coefficient
MSE: Mean Squared Error
MAD: Mean Absolute Deviation
MAPE: Mean Absolute Percentage Error
SSY: Total sum of squares
SSE: Sum of squares of residuals
OOB: Out of Bag
