# Correction to: Multi‑predictor mapping of soil organic carbon in the alpine tundra: a case study for the central Ecuadorian páramo

**DOI:** 10.1186/s13021-021-00198-z

**Published:** 2021-11-15

**Authors:** Johanna Elizabeth Ayala Izurieta, Carmen Omaira Márquez, Víctor Julio García, Carlos Arturo Jara Santillán, Jorge Marcelo Sisti, Nieves Pasqualotto, Shari Van Wittenberghe, Jesús Delegido

**Affiliations:** 1 Image Processing Laboratory (IPL), University of Valencia, 46980 Paterna, Valencia Spain; 2 Faculty of Engineering, National University of La Plata, B1900TAG La Plata, Argentina; 3 Faculty of Engineering, National University of Chimborazo, Riobamba, 060150 Ecuador; 4 Faculty of Forestry and Environmental Sciences, University of Los Andes, Mérida, 5101 Venezuela; 5 Faculty of Science, University of Los Andes, Mérida, 5101 Venezuela; 6 Faculty of Natural Resources, Higher Superior Polytechnic School of Chimborazo, Riobamba, 060155 Ecuador

## Correction to: Carbon Balance Manage (2021) 16:32 https://doi.org/10.1186/s13021-021-00195-2

Following publication of the original article [1], the authors reported errors in the reference citation.

Due to a mistake during the editing process, in the reference numbering of the cited literature, all references from the sentence "Different studies use the visible region considering that soils with higher carbon content have a darker appearance [63] " on page 5 onward refer to the reference number with 1 index number higher.

So [63] refers to the reference [64], this continues until the last reference cited in the text [112] which refers to the listed reference [113]:

[63] → [64].

…

[112] → [113].
